# Doing Everything: Intersections of Gender and Sexual Orientation in Caregiving for a Spouse With Dementia

**DOI:** 10.1093/geroni/igab046.1890

**Published:** 2021-12-17

**Authors:** Toni Calasanti, Brian de Vries

**Affiliations:** 1 VIrginia Tech, Blacksburg, Virginia, United States; 2 San Francisco State University, Palm Springs, California, United States

## Abstract

Gender inequalities are rooted in and drive the division of labor over the life course, which result in heterosexual men and women acquiring different resources, skills, and identities. Gendered differences in caregiving reflect these varying gender repertoires. Whether and how these repertoires vary by sexual orientation is lesser understood. Our qualitative study seeks to explore the ways that sexual orientation and gender, and the related division of both paid and unpaid labor, shapes caregiving for a spouse or partner with Alzheimer’s disease and related disorders (AD). Our data, obtained from in-depth interviews conducted among lesbian (n=9), gay (n=6), and heterosexual spousal and partner (23 women and 14 men) caregivers of those with AD, reveal that, although all the caregivers spoke about “having to do everything,” with a particular focus on decision-making, they interpret this experience differently based on the intersections of gender and sexuality. The heterosexual women reported they were used to managing daily household life, yet they described having to make decisions as quite stressful: “I don’t like to be the boss.” Heterosexual husbands also lamented that they “had to do everything,” but commenting that they hadn’t realized what it took to “manage a household.” The concerns reported by lesbian and gay spouses and partners were similarly situated but more varied, as each group tended to report their previous divisions of labor as “less well-defined.” Our findings reflect both the influence of gender inequalities on how respondents experience “doing everything,” and their potential modification in same-sex relationships.

